# How to predict the existence of epicardial conduction in the right pulmonary vein carina

**DOI:** 10.1002/joa3.12973

**Published:** 2023-12-08

**Authors:** Naoya Kataoka, Teruhiko Imamura

**Affiliations:** ^1^ Second Department of Internal Medicine University of Toyama Toyama Japan

To editor:

Nehashi et al. conducted an investigation into the anatomical indicators associated with the presence of epicardial conduction toward the right pulmonary vein (RPV) carina. The authors presented evidence indicating that anatomical features suggestive of a wider RPV carina region were predictive of the existence of an epicardial connection.^1^ However, several concerns have emerged from their study.

The authors postulated that epicardial conduction toward the RPV carina originated from the right atrium.^1^ Nonetheless, they did not provide demonstrative evidence of a connection between the right atrium and the RPV carina.^2^ Was right atrial mapping performed by the authors to substantiate their hypothesis?

In their study, the authors mentioned that observing high‐voltage PV potential in the RPV carina subsequent to RPV circumferential ablation aided in suspecting the presence of epicardial conduction.^1^ Nevertheless, this observation might not deviate significantly from the conventional procedure, which entails re‐mapping to ascertain the site of earliest activation following RPV circumferential ablation. Instead, an alternative concurrent procedure alongside conventional RPV circumferential ablation ought to be proposed in instances where the RPV carina exhibits high voltage. For instance, supplementary ablation directed at the RPV carina could be recommended subsequent to RPV isolation when the voltage of the RPV carina surpasses predetermined thresholds.

Although the authors hypothesized the existence of epicardial conduction,^1^ it is plausible that alternative conduction pathways toward the RPV carina exist. Notably, a lower contact force has been associated with RPV carina breakthrough after PV ablation.^3^ The actual contact force during PV isolation should be explicitly stated in the authors' study.

The authors explored the implication of anatomical features in patients undergoing circumferential ablation.^1^ PV isolation can be achieved using alternative modalities such as cryoballoon ablation, and RPV carina may also be concurrently ablated using these balloon‐based procedures. The concerns regarding the issue of epicardial connections at the RPV carina may not be problematic in such a procedure. Do the findings of this study extend to other ablation procedures?

## CONFLICT OF INTEREST STATEMENT

The authors declare no conflicts of interest.
